# Assessing the effects of an educational program for the prevention of work-related musculoskeletal disorders among school teachers

**DOI:** 10.1186/1471-2458-14-1211

**Published:** 2014-11-24

**Authors:** Jian Shuai, Pengying Yue, Liping Li, Fengying Liu, Sheng Wang

**Affiliations:** Injury Prevention Research Center, Medical College of Shantou University, 22 Xin Ling Road, Shantou, 515041 China; Xi’an Innovation College of Yan’an University, Xi’an, 710100 China; Guangdong Provincial Center for STI & Skin Diseases Control and Prevention, No.2 Lu Jing Road, Guangzhou, 510095 China; Department of Occupational and Environmental Health, School of Public Health, Peking University, Beijing, 100191 China

**Keywords:** Teachers, Work-related Musculoskeletal Disorders, Intervention

## Abstract

**Background:**

Musculoskeletal disorders represent one of the most common and most costly occupational health problems in both developed and developing countries. The aim of this study was to assess the effect of occupational health education and ergonomic training on awareness, attitude and behavior of work-related musculoskeletal disorders among teachers.

**Methods:**

A self-controlled longitudinal study with pre/post design was used to evaluate the effects of intervention among school teachers from the 21^st^ of June, 2010 to the 21^st^ of August, 2011. Choosing a cluster random sampling method, 350 (70.0% response rate (350/500)) teachers from four schools were assigned to receive eight weeks of intervention (participatory ergonomic training and occupational health education). Evaluations focused on teachers who participated in both pre- and post-questionnaires. Two post-tests were then administered to the participants to identify changes at six and 12 months after intervention.

**Results:**

The follow-up rate was 93.7% (328/350) at six months after intervention, and 90.9% (319/350) at 12 months after intervention. After the intervention, the awareness rate, attitude and health behavior improved. The self-reported prevalence of work-related musculoskeletal disorders for neck, shoulder, upper and lower back pain, or discomfort were lower than before intervention (*P* < 0.05).

**Conclusion:**

Interventions based on occupational health education lectures, on-site ergonomics training, publicity brochures and posters showed a positive effect on prevention and control of the occurrence of work-related musculoskeletal disorders in teachers. Improvement in awareness, behavior and attitude changes, and prevalence were found at both six and 12 months post-intervention, confirming that the effectiveness of the program can be sustained.

## Background

Musculoskeletal disorders represent one of the most common and most costly occupational health problems in both developed and developing countries[1]. With social production highly mechanized, work-related musculoskeletal disorders (WMSDs) are becoming a major health problem encountered by professionals[2–5]. The prevalence of WMSDs linearly correlates with age and length of service[5]. In many industrialized countries, WMSDs has become the second highest occupational disease after occupational mental diseases[6, 7]. Because of the different work characteristics, conditions and working strength, multiple parts of WMSDs are also different[3, 4, 8–10].

Concerns about the risk of WMSDs have been increasing in the education world. School teachers in general, relative to other occupational groups, have a high prevalence of WMSDs[1], with a prevalence of between 45% and 91%[10–15]. WMSDs decrease productivity at work due to sick leave, absenteeism and early retirement[16]. Furthermore, musculoskeletal disorders are also one reason for the early retirement of teachers[16, 17]. Musculoskeletal complaints, especially of the lower back, neck and shoulders, are also common among teachers due to prolonged desk work, prolonged standing in class and repetitive overhead writing on the board, prolonged sitting resulting from frequent reading, preparation of lessons and marking of assignments, and working on a computer[1, 5, 10, 12, 14]. Health education and ergonomics training is an important means of effective prevention and control of musculoskeletal injury[18]. According to Santos et al., aiming a specific educational program toward the prevention of WMSDs is comparable to a general health orientation for the improvement of the quality of life and work capacity in a sample of healthy workers during a six month period[19]. While there is a large published literature, that goes back 10 years or more,[1, 5, 10, 11, 13–17] on the relation between teachers and WMSDs, little has been published on the prevalence of WMSDs, including intervention studies, aimed at the teacher population of China. Our goal is to present evidence-based intervention strategies for school teachers that will assist in ultimately reducing these potentially career-threatening injuries.

## Methods

### Participants

This research was approved by the Ethics Committee of the Medical College of Shantou University. By using a method of random cluster sampling, four schools were selected out of total 1055 schools in Shantou, then the first four schools were chosen as follow: two primary schools, one junior secondary school, and one senior secodary school. Five hundred teachers were randomly recruited from schools in Shantou city, Guangdong province on June 20, 2010, and followed until August 21, 2011. Teachers from each school were selected as participants using the following inclusion criteria: front-line teachers (directly facing the students and teaching in class every day) and being employed in the current school for at least 12 months. The exclusion criteria included employees in administration, design and logistics; temporary teachers, and teachers who taught for less than 1 year. Teachers meeting the inclusion criteria were identified by the management of each school and invited to participate in the study. All participants signed an informed consent form and the study procedures were approved by the Ministry of Education in the districts where the schools were located. Each participant received an incentive for participation in the study.

### Procedure

**Figure 1:**
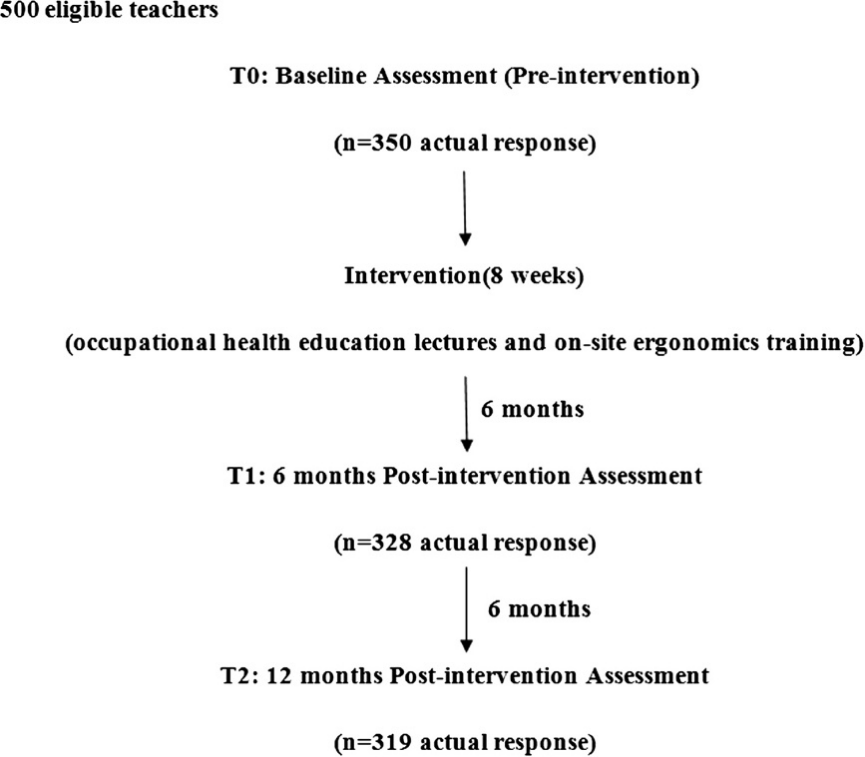
**Time sequence of the study.**

The study consisted of three phases: baseline, intervention, and post-intervention phases during which we had six- and 12-month evaluations, with a follow-up data collection phase at the end of each evaluation to determine the effects of intervention. During the baseline phase, demographics, current physical symptoms, including musculoskeletal pain and work-related personal data (e.g. work posture, knowledge of how to improve posture while working on the computer, including how to minimize strain on forearms, back and neck by adjusting angles and work posture, and how to modify the workstation by changing chair and desk height and backrest inclination, and minimizing hours of computer use) were collected using a specially designed questionnaire (70.0% response rate, 350/500). The participants then entered the intervention phase, in which we implemented eight weeks of intervention by launching a series of occupational health education lectures and on-site ergonomics training. Using the same questionnaire, two post-tests were then administered to the participants to identify changes after intervention. At six months after the intervention phase, we evaluated the effect of intervention through the use of the previous questionnaire (93.7% response rate (328/350)). Following an additional six months, we achieved a 90.9% response (319/350). Data collections were designated as: T0 (baseline for intervention assessment), T1 (six-month post-intervention assessment) and T2 (12-month post-intervention assessment) (Figure 1). The specific interventions are detailed below.

### Questionnaires

A questionnaire based on job specifics of school teachers was designed. The contents of this self-administered questionnaire were constructed and modified from related literature investigating WMSDs among office staff. The design of this domain was in reference to the standardized Dutch Musculoskeletal Questionnaire (DMQ)[20] and standardized Nordic Musculoskeletal Questionnaire (NMQ)[21]. The term “musculoskeletal disorders” here refers to work-related injuries that lasted more than a day, affected daily activities, and happened during work hours. The investigation covered nine body regions, including neck, shoulder, upper back, elbow, hand/wrist, low back, hip/thigh, knee, and ankle/foot. Evaluation of intervention effects included increasing awareness of WMSD-related knowledge, the behavior and attitude, and prevalence rate of WMSDs. Content validity index and inter-rater agreement were examined in previous research[12]. Thirty teachers from another school were surveyed to ensure the construct validity of the questionnaire in the baseline assessment phase. The same group completed the questionnaire again after two weeks. The test–retest reliability of the questionnaire was conducted to demonstrate that the questionnaires were reliable (Kappa 0.83).

### Interventions

The multifaceted intervention comprised of two aspects: 1) an occupational health lecture, approximately 40 min long, introducing musculoskeletal disorders, risk factors, pathogenesis, high-risk groups, and basic ergonomic principles, as well as emphasizing taking breaks and doing exercises while in the office, 2) ergonomic training on how to improve their posture while working on the computer, including recommendations on how to minimize strain on forearms, upper back and neck by adjusting angles and work posture, and practical instruction on how to modify their workstation by changing chair and desk height, backrest inclination, keyboard inclination and location, screen height, inclination and orientation, forearm supports and foot rests as needed. These modifications are supported by the current literature on work space ergonomics[22–27]. In addition, poster foldouts were printed and distributed to remind the school teachers to pay attention to maintaining the recommended correct work posture, taking breaks and doing exercises (a specially designed stretching and strengthening exercise program). The lecture and ergonomics training involved eight weekly sessions[28] for each school by an experienced health educator.

### Statistical analysis

The questionnaires were recorded using EpiData 3.1 software and analyzed with R software (version 3.0.3; R Development Core Team, http://www.r-project.org/). Descriptive statistics were used to identify the frequencies. The proportions of teachers who reported WMSDs in any body part were calculated based on the number of WMSD cases and total respondents. The chi-square test was applied to compare the proportions of teachers. Group comparisons were performed to examine the changes between pre-intervention, six months post-intervention and 12 months post-intervention. A factor loading plot of Correspondence Analysis was fitted using the R package MASS (version 7.3-31). For the factor loading plot of correspondence analysis, we observed the horizontal distance of two points (the group and the answer); the nearer the distance between two points, the closer the relationship between them. The level of significance was set at 0.05. All *P* values are two-sided, and a *P* value less than 0.05 was considered statistically significant in order to balance between type I and type II errors.

## Results

### Effects of intervention on WMSDs-related knowledge among school teachers

Statistical significance revealed between the pre- and post-intervention on the awareness rate of WMSDs related knowledge (*P* < 0.001, Table 1). The frequency of correct answers to “1. What kind of disease is a WMSD?”, “2. Can WMSDs be prevented or controlled?”, “4. Do you know how to adjust the height of the office chair to make yourself more comfortable?”, “9. Should you not leave space, when working in a sitting position, between the seat front and back of your legs?”, “10. If you have neck symptoms, should you raise the pillow to sleep?”, and “11. Is the optimal chair height up to the position of knee?” monotonically increased (*P* < 0.05), indicating that awareness rate of WMSDs related knowledge have improved. The frequency of correct answers to “3. Do you know about physiological bending of the spine?”, “6. Do you know the correct posture for working at your desk?”, and “7. What is the optimal elbow angle for typing at a computer?” markedly departed from monotonicity, with a sharp downward turn at 12 months post-intervention (*P* < 0.01). That awareness of WMSD-related knowledge increased at six months post-intervention, and declined at 12 months post-intervention, indicated that intervention for “physiological bending of the spine”, “correct posture for working at your desk”, and “optimal elbow angle for typing at a computer” had a short-term effect. However, the frequency of correct answers to “5. Do you know the correct posture to use at the computer?” and “8. What is the optimal angle between monitor and your sight line when using a computer?” were close to non-monotonic between six and 12 months post-intervention, showing that there was no significance before and after intervention (*P* > 0.05).

**Table 1:** **Pre- and post-intervention comparative results on WMSD-related knowledge**

Questions	Pre-intervention		Six months post-intervention		12 months post-intervention		***χ*** ^***2***^	***P***
	n	%	n	%	n	%		
**1. What kind of disease is a WMSD?**							143.8128	<0.0001
Correct	89	25.43	156	47.56	229	71.79		
Wrong	261	74.57	172	52.44	90	28.21		
**2. Can WMSDs be prevented or controlled?**							25.2716	<0.0001
Correct	245	70.00	261	79.57	274	85.89		
Wrong	105	30.00	67	20.43	45	14.11		
**3. Do you know about physiological bending of the spine?**							97.7663	<0.0001
Yes	71	20.29	182	55.49	95	29.78		
No	279	79.71	146	44.51	224	70.22		
**4. Do you know how to adjust the height of the office chair to make yourself more comfortable?**							15.7876	0.0004
Yes	73	20.86	107	32.62	105	32.92		
No	277	79.14	221	67.38	214	67.08		
**5. Do you know the correct posture to use at the computer?**							27.5894	<0.0001
Yes	117	33.43	172	52.44	154	48.28		
No	233	66.57	156	47.56	165	51.72		
**6. Do you know the correct posture for working at your desk?**							50.1483	<0.0001
Yes	163	46.57	240	73.17	180	56.43		
No	187	53.43	88	26.83	139	43.57		
**7. What is the optimal elbow angle for typing at a computer?**							92.8680	<0.0001
Correct	61	17.43	170	51.83	95	29.78		
Wrong	289	82.57	158	48.17	224	70.22		
**8. What is the optimal angle between monitor and your sight line when using a computer?**							24.9632	<0.0001
Correct	141	40.29	164	50.00	148	46.39		
Wrong	209	59.71	164	50.00	171	53.61		
**9. Should you not leave space, when working in a sitting position, between the seat front and back of your legs?**							81.4197	<0.0001
Correct	201	57.43	263	80.18	275	86.21		
Wrong	149	42.57	65	19.82	44	13.79		
**10. If you have neck symptoms, should you raise the pillow to sleep?**							156.8531	<0.0001
Correct	218	62.29	295	89.94	309	96.87		
Wrong	132	37.71	33	10.06	10	3.13		
**11. Is the optimal chair height up to the position of knee?**							54.0184	<0.0001
Correct	244	69.71	271	82.62	293	91.85		
Wrong	106	30.29	57	17.38	26	8.15		

### Behavior and attitude changes

**Figure 2:**
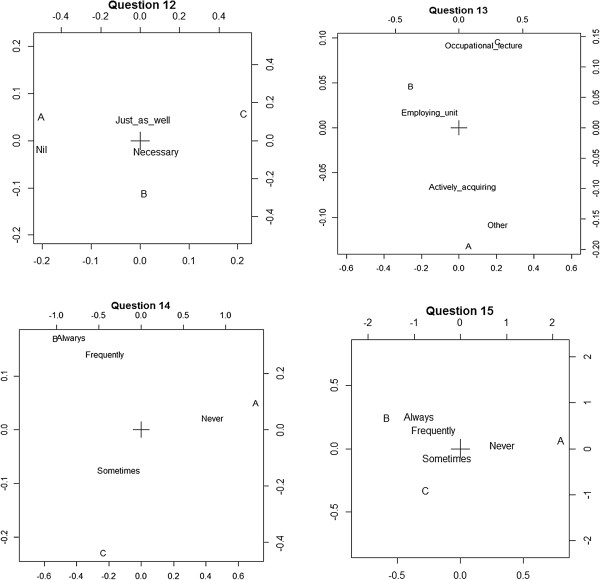
**The factor loading plot of correspondence analysis.** *(* ***A*** *: pre-intervention;* ***B*** *: Six months post-intervention;* ***C*** *: 12 months post-intervention).*

Six months after intervention, there was a significant change in healthy behavior. Teachers paid more attention to keeping an optimum posture, and increased the frequency of stretching exercises performed during work following the intervention. The desire to obtain further knowledge of preventing chronic cumulative musculoskeletal injury also underwent a change (Table 2). The correspondence analysis factor loading plot indicated intervention was effective in “12. What do you think of the necessity to hold disease knowledge lectures and training activities?”, “13. What do you think is the optimal way to acquire prevention and control knowledge of disease?”, “14. Would you pay special attention to keeping the optimal posture at work?”, and “15. Would you ever do some extra stretching exercises during work?” (Figure 2). Assessments of the positive effects “16. How long do you think the positive effects of the occupational health education lectures and ergonomics training can last?”, at six and 12 months after intervention, were identical, showing that the behavior and attitude for prevention of the disease not only improved, but also persisted unabated for at least 12 months (Table 2).

**Table 2:** **Pre- and post-intervention comparison on the action of teachers toward WMSDs**

Questions	Pre-intervention		Six months post-intervention		12 months post-intervention		***χ*** ^***2***^	***P***
	n	%	n	%	n	%		
**12. What do you think of the necessity to hold disease knowledge lectures and training activities?**							34.6124	<0.0001
Necessary	168	48.00	198	60.36	187	58.62		
Just as well	127	36.29	96	29.27	121	37.93		
Nil	55	15.71	34	10.37	11	3.45		
**13. What do you think is the optimal way to acquire prevention and control knowledge of disease?**							45.9329	<0.0001
Employing unit	113	32.29	155	47.26	88	27.59		
Actively acquiring	125	35.71	94	28.66	94	29.47		
Occupational lecture	77	22.00	66	20.12	108	33.85		
Other	35	10.00	13	3.96	29	9.09		
**14. Would you pay special attention to keeping the optimal posture at work?**							314.1653	<0.0001
Never	209	59.71	19	5.79	51	15.99		
Sometimes	106	30.29	183	55.79	210	65.83		
Frequently	35	10.00	109	33.24	51	15.99		
Always	0	0	17	5.28	7	2.19		
**15. Would you ever do some extra stretching exercises during work?**							429.6923	<0.0001
Never	230	65.71	19	5.79	45	14.11		
Sometimes	100	28.57	183	55.79	232	72.73		
Frequently	20	5.72	109	33.23	39	12.23		
Always	0	0	17	5.19	3	0.94		
**16. How long do you think the positive effects of the occupational health education lectures and ergonomics training can last?**							4.4388	0.2178
Not a bit	-	-	58	17.68	77	24.14		
1 week	-	-	105	32.01	100	31.35		
30 days	-	-	84	25.61	74	23.20		
The entire semester	-	-	81	24.70	68	21.32

### Effects of intervention on WMSD prevalence

**Figure 3:**
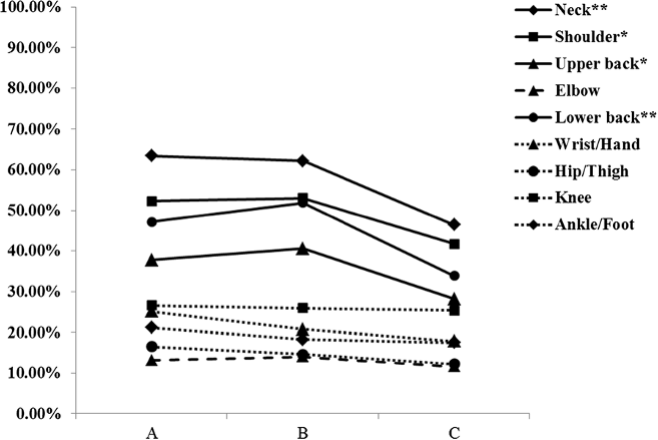
**Tendency of the prevalence of WMSDs changes pre- and post-intervention.** (***A*** *: pre-intervention;* ***B*** *: Six months post-intervention;* ***C*** *: 12 months post-intervention*; *: *P* < 0.01; **: *P* < 0.001).

The self-reported WMSD prevalence for neck, shoulder, upper and lower back pain or discomfort, was lower at 12 months post-intervention than at either pre-intervention or six months post-intervention (*P* < 0.05, Table 3). However, the self-reported WMSD prevalence for elbow, wrist/hand, hip/thigh, knee, and ankle/foot pain or discomfort was not significantly different between pre- and post-intervention (*P* > 0.05, Table 3, Figure 3).

**Table 3:** **Comparative pre- and post-intervention results on prevalence of WMSDs**

Places	Pre-intervention		Six months post-intervention		12 months post-intervention		***χ*** ^***2***^	***P***
	n	%	n	%	n	%		
Neck	222	63.43	204	62.20	148	46.40	24.099	0.000
Shoulder	183	52.29	174	53.05	133	41.69	10.470	0.005
Upper back	132	37.71	133	40.55	90	28.21	11.777	0.003
Elbow	46	13.14	46	14.02	37	11.60	0.864	0.649
Lower back	165	47.14	170	51.83	108	33.86	22.763	0.000
Wrist/hand	88	25.14	68	20.73	57	17.87	5.373	0.068
Hip/thigh	58	16.57	48	14.63	39	12.22	2.539	0.281
Knee	93	26.57	85	25.91	81	25.39	0.122	0.941
Ankle/foot	74	21.14	60	18.29	56	17.55	1.578	0.454

## Discussion

WMSDs have become a large concern in occupational health and are expected to increase in both prevalence and severity because of the changing nature of work and the aging of the workforce. WMSDs affect a large number of employees every year. In some countries, intervention studies in prevention and control of WMSDs have been carried out and achieved positive results[29–31], but research is lacking on corresponding intervention among teachers in China. Compared with the developed countries, Chinese teachers comprise a relevant occupational group because the population of China is large. In order to alleviate the heavy work load and pressure, seeking simple and effective intervention measures is significant. Prior research shows that WMSD risk factors for teachers include: a total class time of more than 14 hours per week, sitting or standing for a long time, maintaining the same position or twisting the body, and lack of facilities on which to lean[12, 13, 32, 33]. Based on these risk factors, we performed intervention measures consisting of occupational health education and ergonomics training. Before the intervention, teacher understanding of musculoskeletal injury or disease was unclear and not systematic. Although most teachers realize that disease can be prevented, they are unclear about prevention techniques. After the intervention, the level of understanding of disease, health attitudes and behavior, and reductions in annual prevalence of injury greatly improved.

### Awareness of WMSD-related knowledge

The increase in correct answers, following intervention, closely follows a monotonic increase, indicating that the effects of these aspects are sustained through the intervention. That the frequency of correct answers for other aspects markedly departs from monotonicity indicates intervention has a short-term effect. However, there is no significance before and after intervention regarding posture (“Do you know the correct posture to use at the computer?” and “What is the optimal angle between monitor and your sight line when using a computer?”). Shantou City is a low educational level area in China. Teachers teach multiple courses and have no time to learn specialized knowledge, such as physiological bending of the human spine, correct posture to use at a computer, correct posture for working at a desk, optimal elbow angle for typing at a computer, or optimal angle between monitor and sight line when using a computer. This would indicate that long-term specialized training for WMSD-related prevention should be considered in the future. WMSDs are chronic cumulative occupational injuries. Teachers need long-term cumulative formation and reinforcement of proper habits to change their behavior, as subject initiative plays an important role.

### Behavioral and attitude changes

Ergonomics training makes teachers pay special attention to maintaining optimal posture while working at a desk for long periods. After our intervention, behavior and attitude toward prevention and control of musculoskeletal injury improved. Educational lectures on occupational health enables teachers to adjust their own work schedule to incorporate breaks and time for stretching exercises, which can reduce psychological pressure and the static pressure load of individuals, and have the greatest impact on preventing and controlling WMSDs. da Costa provided mixed findings, but demonstrated some beneficial effects of stretching exercises in preventing WMSDs[34]. With prolonged desk work and computer use, teachers are similar to office staff. Long-term use of computers is associated with various musculoskeletal disorders, and exercise and posture correction alleviate or reduce these disorders[25]. The effectiveness of ergonomic training is verified both visually and objectively. Robertson et al. revealed that participants who receive ergonomics training have minimal musculoskeletal and visual discomfort, over the workday, and higher performance compared to the control group, suggesting that a comprehensive training program can play a significant role[35]. In addition, Robertson suggests that the provision of ergonomic skills allows individuals to make appropriate workstation changes, thus reducing musculoskeletal risks and discomfort associated with computer work, and improving organizational effectiveness[23]. Similarly, CBT (cognitive behavioral therapy) is one of the most effective ways to prevent and control the occurrence of low back pain[36].

### Prevalence change within 12 months

Prevalence of WMSDs, over the 12 months following intervention, declines in the neck, shoulder, low back and waist, where teachers mainly suffer from pain and discomfort of WMSDs[12]. These locations of musculoskeletal injury are related to the nature of the work (e.g. heavy prolonged desk work load and pressure) in teachers. The prevalence of neck injury declines after intervention, but the prevalence of shoulder, upper and lower back pain takes a marked departure from monotonicity, with a moderate downward turn at 12 months post-intervention. It is likely that the self-reported frequency depends increasingly on the awareness of WMSDs after intervention. Yu et al. showed that there are significance reductions in WMSDs of the lower extremities, wrist and fingers in manufacturing workers after training[37]. Musculoskeletal complaints, such as for ankle/foot and wrist/hand, are common among manufacturing workers due to operation of machines through the use of lower extremities, wrist and fingers. The difference is due to the characteristics of the occupation.

Schools vary by scale, degree, nature, and teaching content. Randomization of small numbers of clusters (schools) may not adequately deal with potential confounding factors (e.g. contamination in the workplace). Although the education of teachers is higher, it is still difficult to avoid some information bias. Recall bias of the participant impedes our ability to compare post-intervention knowledge to baseline (pre-intervention), as well as our ability to assess the effect of the intervention. However, the design of the present study could be improved. Our study was pre/post designed without a control group so that contamination could not be adequately examined. Due to the lack of information on gender, confounding bias was not effectively controlled. Despite this, there are potential applications of this intervention model for teachers in other countries, and the intervention can also be modified to be implemented in schools for prevention and control of WMSDs. With the development of WMSD-related studies, new methods to evaluate the WSMDs are required[38–42]. Further high quality studies, increasing the number of observations over time, or extending the length of the study, are needed to support evidence-based ergonomic interventions in practice[37, 43, 44].

## Conclusions

This study provides evidence on the effectiveness of a multifaceted ergonomic intervention program designed to reduce musculoskeletal symptoms in teachers. With increasing work pressure, it is critical that teachers become more aware of the risk of WMSDs and learn ways to minimize such disorders for their own well-being. Interventions based on occupational health education lectures, on-site ergonomics training, publicity brochures and posters have a positive effect on preventing and controlling the occurrence of WMSDs in teachers.

## Author’s information

First author: Jian Shuai.

## Abbreviations

WMSDs: Work-related musculoskeletal disorders
DMQ: Dutch musculoskeletal questionnaire
NMQ: Nordic musculoskeletal questionnaire.
